# Interaction between Maternal and Offspring Diet to Impair Vascular Function and Oxidative Balance in High Fat Fed Male Mice

**DOI:** 10.1371/journal.pone.0050671

**Published:** 2012-12-05

**Authors:** Christopher Torrens, Priya Ethirajan, Kimberley D. Bruce, Felino R. A. Cagampang, Richard C. M. Siow, Mark A. Hanson, Christopher D. Byrne, Giovanni E. Mann, Geraldine F. Clough

**Affiliations:** 1 Human Development & Health, Faculty of Medicine, University of Southampton, Southampton, United Kingdom; 2 Nutrition and Metabolism, University of Southampton, Southampton, United Kingdom; 3 Southampton National Institute for Health Research Biomedical Research Centre, Southampton General Hospital, Southampton, United Kingdom; 4 Cardiovascular Division, BHF Centre of Research Excellence, School of Medicine, King’s College London, London, United Kingdom; State University of Rio de Janeiro, Biomedical Center, Institute of Biology, Brazil

## Abstract

**Aims:**

To determine the impact of maternal and post-weaning consumption of a high fat diet on endothelium-dependent vasorelaxation and redox regulation in adult male mouse offspring.

**Methods:**

Female C57BL6J mice were fed an obesogenic high fat diet (HF, 45% kcal fat) or standard chow (C, 21% kcal fat) pre-conception and throughout pregnancy and lactation. Post-weaning, male offspring were continued on the same diet as their mothers or placed on the alternative diet to give 4 dietary groups (C/C, HF/C, C/HF and HF/HF) which were studied at 15 or 30 weeks of age.

**Results:**

There were significant effects of maternal diet on offspring body weight (*p*<0.004), systolic blood pressure (*p* = 0.026) and endothelium-dependent relaxation to ACh (*p* = 0.004) and NO production (*p* = 0.005) measured in the femoral artery. With control for maternal diet there was also an effect of offspring post-weaning dietary fat to increase systolic blood pressure (*p*<0.0001) and reduce endothelium-dependent relaxation (*p* = 0.022) and ACh-mediated NO production (*p* = 0.007). There was also a significant impact of age (*p*<0.005). Redox balance was perturbed, with altered regulation of vascular enzymes involved in ROS/NO signalling.

**Conclusions:**

Maternal consumption of a HF diet is associated with changes in vascular function and oxidative balance in the offspring of similar magnitude to those seen with consumption of a high fat diet post-weaning. Further, this disadvantageous vascular phenotype is exacerbated by age to influence the risk of developing obesity, raised blood pressure and endothelial dysfunction in adult life.

## Introduction

Over weight and obesity are associated with an increased risk of cardiovascular and metabolic disease, affecting all ages and socioeconomic groups [1]. The prevalence of childhood obesity has increased dramatically in recent decades. Overweight children prematurely develop vascular endothelial dysfunction, hypertension and type 2 diabetes normally found in older obese adults [2]. An increased prevalence of childhood obesity correlates with the increase in the number of overweight and obese women becoming pregnant [3] and there are now well recognised consequences for the long term health of the children born to pregravid over weight and obese mothers [4].

Obesity is frequently related to an excessive long-term intake of fats and chronic exposure to a high fat diet is associated with an impaired endothelium-dependent vasodilation and altered oxidative balance [5], [6]. While low levels of reactive oxygen species (ROS) play a physiological role in cell signalling and vascular function [7], enhanced production or diminished scavenging of these radicals may lead to a reduction in nitric oxide (NO) bioavailability and/or endothelial nitric oxide synthase (eNOS) uncoupling and increased formation of superoxide that are associated with impaired vascular function [8]–[10]. The protective role of endogenous antioxidant mechanisms in cardiovascular disease and their disturbance in obesity related disorders is less clearly defined [7].

Studies in animal models have shown that maternal over-nutrition and obesity during pregnancy predispose offspring to adiposity, hypertension and insulin resistance [11]–[15] with changes in regulation of energy balance in key metabolic tissues such as skeletal muscle and in adipocyte metabolism playing a role in the development of adiposity and increased risk of cardiovascular dysfunction. Maternal high fat feeding also predisposes to non-alcoholic fatty liver disease (NAFLD) in adult offspring, associated with hepatic mitochondrial dysfunction and altered hepatic oxidative stress [16]–[18] and antioxidant defence capacity [19]. However, the mechansisms underlying the development of an adverse vascular phenotype remain uncertain and the prenatal priming of impaired vascular redox regulation in offspring of dams fed a high fat diet has yet to be fully investigated [20].

**Table 1:** Dietary composition of macronutrients and energy values in standard laboratory chow diet and high fat diet used in this study.

	Chow Diet	High Fat Diet
Percentage in weight (gm)		
Carbohydrate	70.0	49.5
Protein	18.0	26.5
Lipid	10.0	22.5
Percentage in energy (kcal)		
Carbohydrate	62.9	35.0
Protein	16.5	20.0
Lipid	20.6	45.0
Fat breakdown (Mole % FA)		
Saturated FA’s	24.8	41.5
Monounsaturated FA’s	38.0	37.9
Polyunsaturated FA’s	37.2	20.6

Abbreviations: FA: fatty acids.

The present study was designed to investigate the impact of a maternal high fat diet pre-conception and during pregnancy and suckling on endothelium-dependent vasorelaxation in adult male mouse offspring and to determine whether dysregulation of redox balance plays a mechanistic role in influencing such vascular reactivity. We set out to test whether the vascular phenotype of adult mouse offspring of dams fed a high fat diet during pregnancy, and suckling and then maintained on a standard chow diet, was comparable with that of mice fed a high fat diet only post-weaning, and if it was exacerbated in offspring maintained on the same high fat diet as their dams in adult life - a ‘double hit’. Furthermore, offspring were studied at two ages, 15 and 30 weeks, in order to explore the impact of age. We assessed endothelium-mediated vascular relaxation in the femoral artery by measuring the response to acetylcholine (ACh). NO production was assessed using 4,5-diaminofluorescein diacetate (DAF-FM), and indices of oxidative stress using the redox-sensitive dye dihydroethidium (DHE) and expression of nicotinamide adenine dinucleotide phosphate-oxidase 2 (Nox2), a subunit of NADPH oxidase and the stress response enzyme heme oxygenase-1 (HO-1).

**Figure 1:**
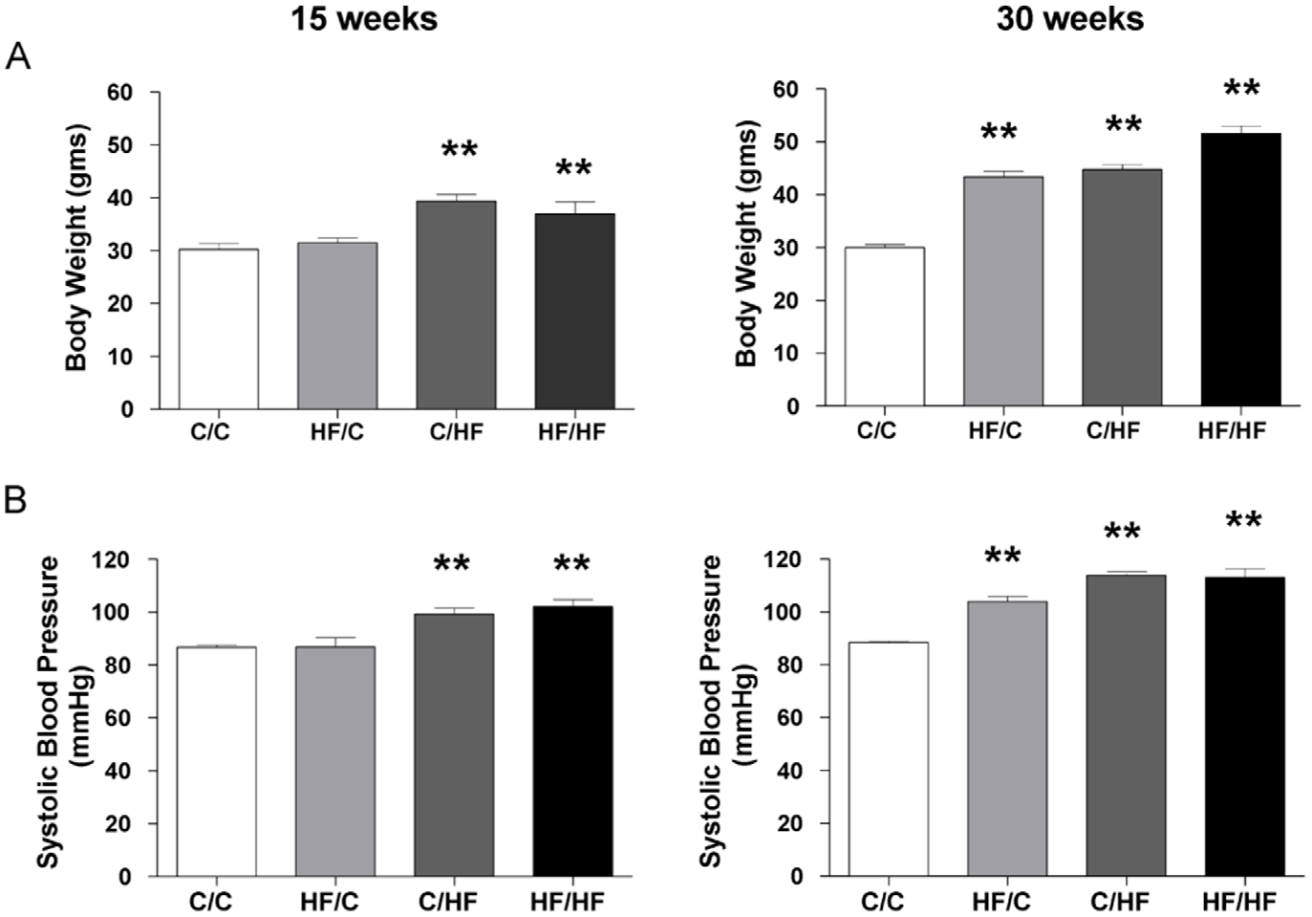
Phenotype of male mouse offspring at 15 and 30 weeks of age. Dams were assigned to either a high fat (HF) diet or standard chow (C) for 4 weeks before conception and during gestation and lactation. At weaning, offspring were assigned to C or HF to give four dietary groups C/C, HF/C, C/HF and HF/HF. Bar graphs represent mean ± SEM for (A) Body weight from 15 week C/C n = 9, HF/C n = 8, C/HF n = 7, HF/HF n = 9 and 30 week C/C n = 10, HF/C n = 10, C/HF n = 10, HF/HF n = 10 offspring, and (B) Systolic blood pressure measured by tail-cuff plethysmography from 15 week and 30 week offspring (C/C n = 7, HF/C n = 7, C/HF n = 5, HF/HF n = 9). Statistical comparisons were by ANOVA for the effects of maternal and offspring diet and age (see Table 2) followed by analysis using *post hoc* Dunnett’s multiple comparison tests for HF/C, C/HF and HF/HF vs. C/C. Values significantly different between high fat fed offspring groups and control offspring (C/C) at 15 or 30 weeks of age are indicated by ** *p*<0.001.

**Table 2:** Effects of maternal high fat diet, post-weaning high fat diet and offspring age on phenotypic and biochemical factors in 15 and 30 week old offspring as determined by multiple ANOVA analysis.

Variable	Body weight	Systolic blood pressure	ACh-mediated relaxation in femoral artery	Resting NO production in femoral artery	ACh-induced NO production in femoral artery	ROS femoral artery
**Maternal diet**	F = 8.9 *p* = 0.004	F = 5.3 *p* = 0.026	F = 8.9 *p* = 0.004	F = 6.4 *p* = 0.021	F = 10.1 *p* = 0.005	F = 3.8 *p* = 0.064
**Offspring diet**	F = 3.01 *P* = 0.087	F = 65.3 *p*<0.0001	F = 5.6 *p* = 0.022	F = 8.5 *p* = 0.009	F = 9.2 *p* = 0.007	F = 15.5 *p* = 0.001
**Age**	F = 24.8, *p*<0.0001	F = 32.8 *p*<0.0001	F = 34.0 *p*<0.0001	F = 9.9 *p* = 0.005	F = 11.1 *p* = 0.003	

## Materials and Methods

### Materials

The animal diets were from Special Diet Services UK. Acetylcholine (ACh), Norepinephrine (NE), Sodium Nitroprusside (SNP) and N^ω^-nitro-L-arginine methyl ester (L-NAME) were from Sigma-Aldrich (UK); DAF-FM from Invitrogen (UK) and DHE from Invitrogen (UK). Antibodies against NADPH oxidase 2 (Nox2) and hemoxygenase-1 (HO-1) were from Santa Cruz Biotechnology (USA), α-tubulin was from Santa Cruz Biotechnology (USA) and the HRP-conjugated anti-goat secondary antibody was from Millipore (UK). The enhanced chemiluminescence detection reagent was from Thermo Scientific (UK).

**Figure 2:**
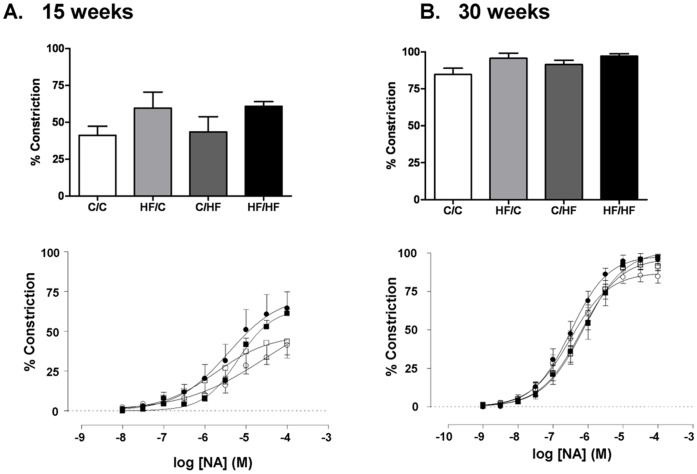
Impact of maternal HF-feeding on offspring vasoconstrictor responses in the femoral artery. Cumulative dose response curves and maximum response (%) to the vasoconstrictor noradrenaline (NA) measured in the femoral artery of male mice offspring from four dietary groups at (A) 15 weeks of age and (B) 30 weeks of age C/C (○) H/FC (•) C/HF (□) or HF/HF (▪). Data are mean ± SEM (n = 6 per group).

**Figure 3:**
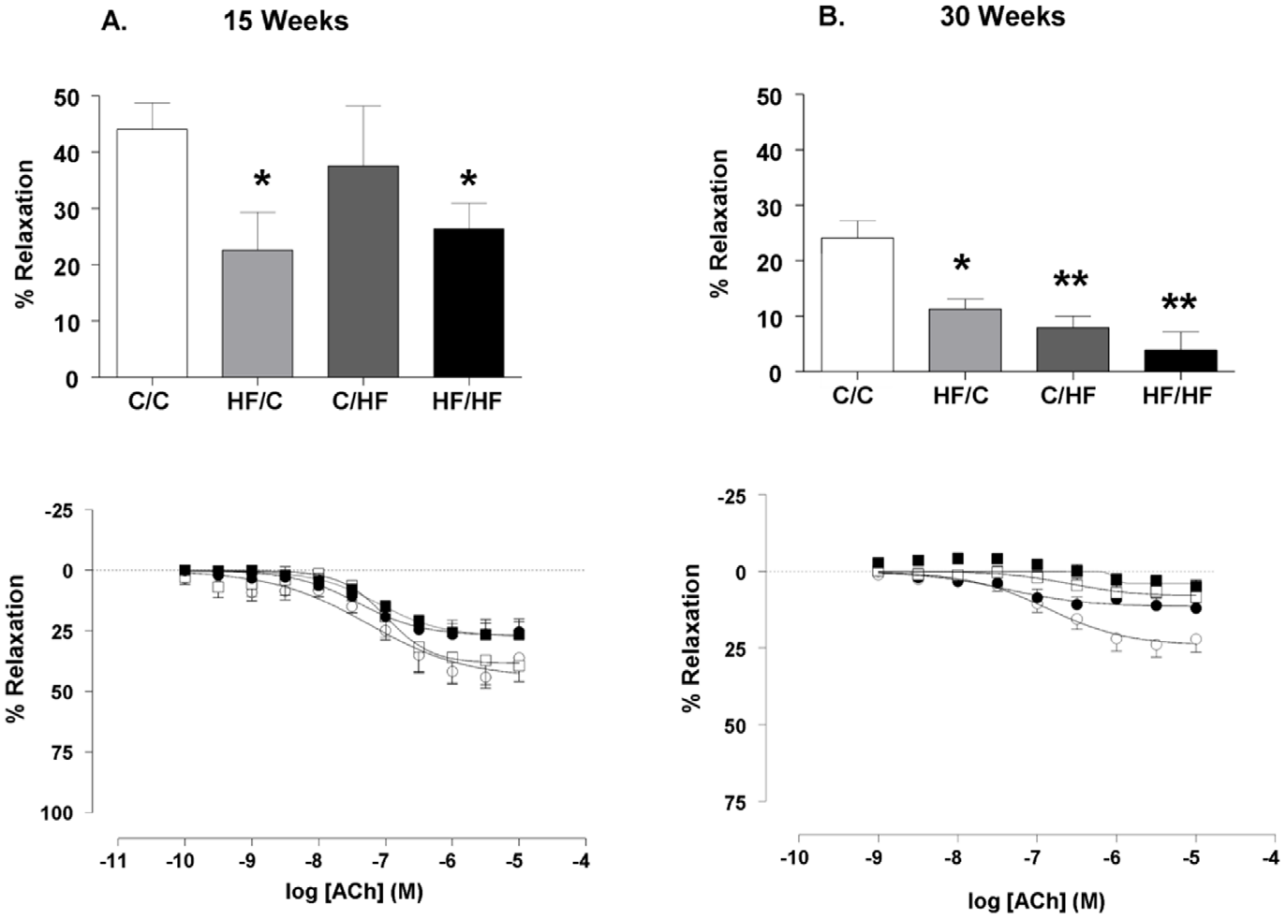
Impact of maternal HF-feeding on offspring vasorelaxation in the femoral artery. Cumulative dose response curves and % maximum response to the vasodilator (ACh) measured in the femoral artery of male mice offspring from four dietary groups at (A) 15 weeks (○ C/C n = 6, • HF/C n = 7, □ C/HF n = 6, ▪ HF/HF n = 7) and (B) 30 weeks of age. (○ C/C n = 8, • HF/C n = 6, □ C/HF n = 8, ▪ HF/HF n = 7). Data are mean ± SEM. Values significantly different between high fat fed offspring groups and control offspring (C/C) are indicated by * *p*<0.05, **p<0.001.

### Ethical Statement

All animal procedures were in accordance with the regulations of the United Kingdom Animals (Scientific Procedures) Act 1986 and were conducted under Home Office Licence number 70-6457. The study received institutional approval from the University of Southampton Biomedical Research Facility Research Ethics Committee.

**Figure 4:**
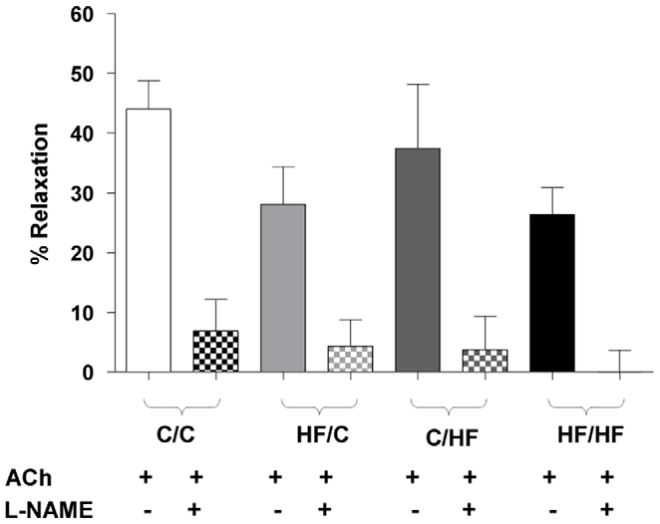
Impact of maternal HF-feeding on offspring NO-component of the ACh-induced vasorelaxation. Femoral dilation to ACh alone (open columns) or in the presence of L-NAME (100 µM) (shaded columns) in the four male offspring groups at 15 weeks of age. Within offspring group statistical comparisons were made using a paired t test. Data are mean ± SEM, (n = 4–6 per group). * *p*<0.01.

**Figure 5:**
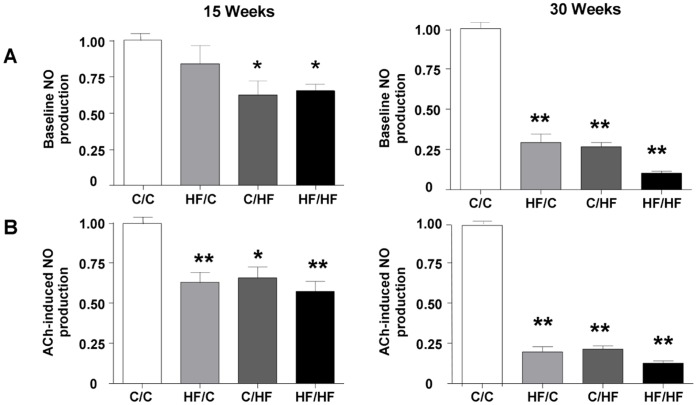
Impact of maternal HF-feeding on offspring NO production in femoral artery. (A) Basal NO production and (B) ACh-stimulated NO production in femoral arteries as detected by using 4,5-diaminofluorescein diacetate (DAF-FM) normalised to NO production by C/C offspring group (n = 3 per group). Data are expressed as mean ± SEM. Statistical comparisons were by ANOVA for the effects of maternal and offspring diet and age followed by comparisons using Dunnett’s Multiple Comparison Test for HF/C, C/HF and HF/HF vs. C/C. Values significantly different between high fat fed offspring groups and control offspring (C/C) at 15 or 30 weeks of age are indicated by * *p*<0.05, **p<0.01. (Please see *Figure S4* for confocal images of DAF staining in the four offspring groups.).

**Figure 6:**
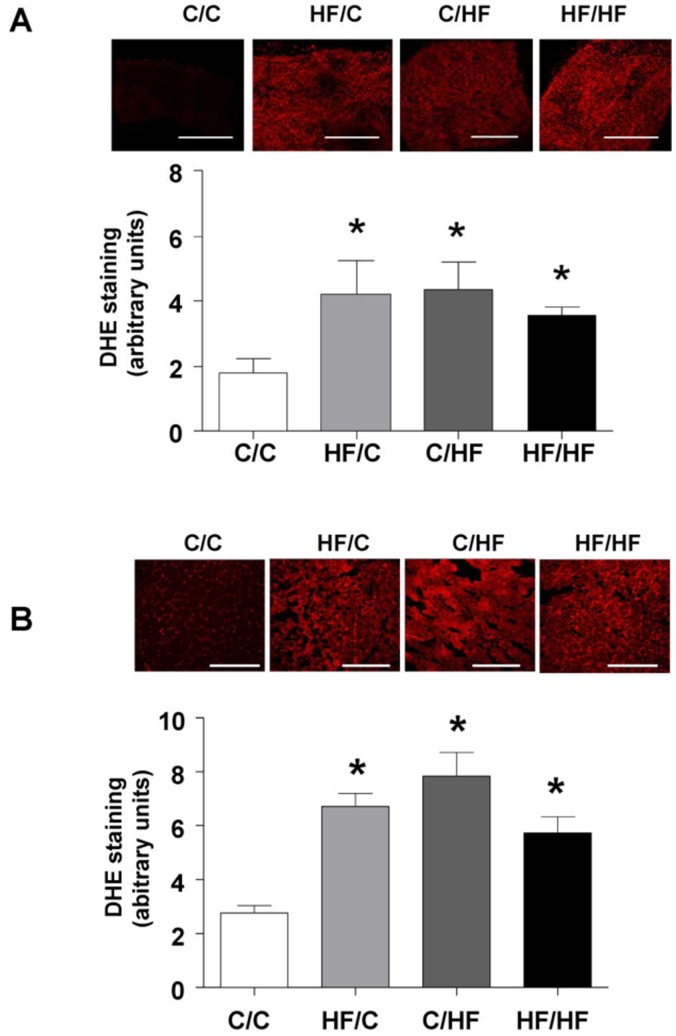
Impact of maternal HF-feeding on offspring redox regulation. Superoxide generation in (A) fresh femoral artery segments from 15 weeks (n = 4 per group) and (B) vastus muscle from 30 week old offspring (n = 5 per group) assessed using the redox-sensitive dye dihydroethidium (DHE) staining (5 µM in PBS). Representative confocal images obtained from femoral artery and vastus segments from the four offspring groups are shown above each bar graph. Scale bar = 250 µm. Bar graphs represent mean ± SEM. Statistical comparisons were by ANOVA for the effects of maternal and offspring diet followed by comparisons using Dunnett’s Multiple Comparison Test for HF/C, C/HF and HF/HF vs. C/C. * *p*<0.05).

**Figure 7:**
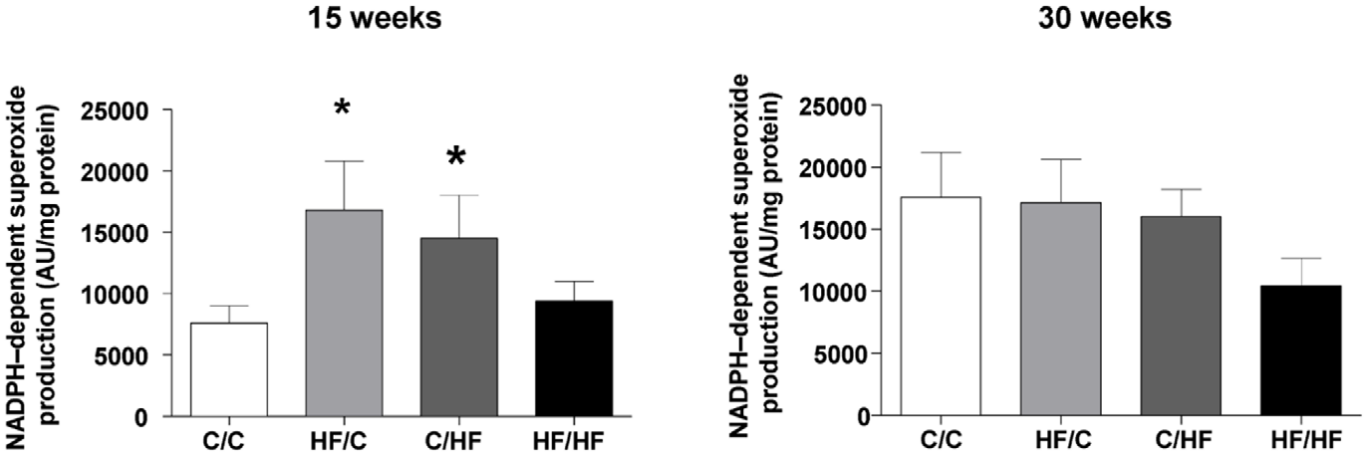
Impact of maternal HF-feeding on offspring redox regulation. in the liver. NADPH activity in liver homogenates estimated using a lucigenin chemiluminescence assay. Values are expressed as mean light units (AU) per mg protein. Each determination was performed in triplicate and results obtained were normalized for total protein. Bar graphs represent mean ± SEM from n = 6 per group.

**Figure 8:**
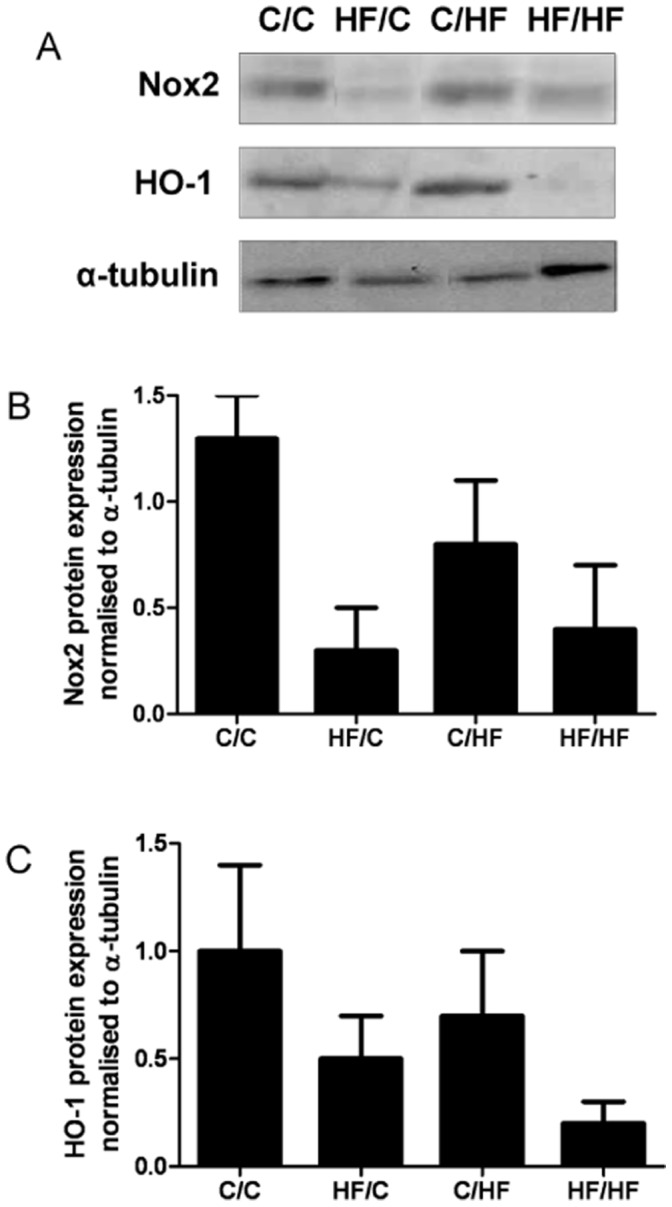
Impact of maternal HF-feeding on offspring Nox2 and HO-1 protein expression. A, Representative immunoblots of Nox2, HO-1 and α-tubulin. B, Densitometric analyses of Nox2 and HO-1 relative to α-tubulin and normalised to C/C in aortic tissue lysates from 30 week old male offspring from the four dietary groups. Data are mean ± SEM from C/C n = 5, HF/C n = 5 C/HF n = 5, HF/HF n = 4 offspring per group.

**Figure 9:**
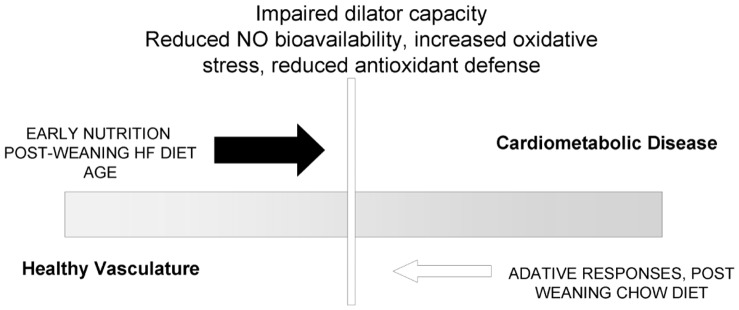
Schematic of processes by which the pre-natal environment ‘primes’ for vascular dysfunction in the offspring.

### Animal Procedures

Female C57BL/6J mice (n = 20) were fed either standard chow (C, 21% kcal fat) or saturated fat (HF, 45% kcal fat) (Table 1) for 4 weeks prior to conception, during gestation and lactation as previously described [18]. (Please see online supplement for dietary model *Figure S1*). Maternal body weight was determined on commencing the study and immediately prior to conception. At term dams were allowed to deliver and litters were standardized to 6 pups. At 21 days of age offspring were weaned onto either a HF or C diet, generating 4 experimental groups: C/C, C/HF, HF/C and HF/HF. Male offspring were studied at either 15 or 30 weeks of age. The dams were killed following weaning of their offspring. Their body weight and the weights of the different fat pads, consisting of the intrascapular, retroperitoneal, inguinal, perirenal and gonadal fat, were measured. The percent of body weight accounted for as fat was calculated. To avoid maternal bias two sets of dams were used to generate the 15 and 30 week old male offspring. At a given age point, no more than two offspring per dam were studied and where a given variable was measured in two offspring from the same litter, measurements from the two offspring were treated as replicates and averaged. At 15 and 30 weeks of age, male offspring were killed by an overdose of anaesthetic (isofluorane) and cervical dislocation. The various fat pads were dissected and weighed (as described above). Femoral arteries were prepared for functional response, imaging and gene and protein expression studies. Skeletal muscle (vastus lateralis) and liver (left lobe) were dissected for imaging or snap-frozen in liquid nitrogen.

### Evaluation of Blood Pressure and Vascular Function

Offspring systolic blood pressure was measured at 15 and 30 weeks by tail-cuff plethysmography as previously described [21]. Vascular function was assessed post mortem in femoral arteries using wire myography as previously described [21]. Briefly, femoral arteries were dissected and stored in cold (4°C) physiological salt solution (PSS) of the following composition; NaCl, 119; KCl, 4.7; CaCl_2_, 2.5; MgSO_4_, 1.17; NaHCO_3_, 25; KH_2_PO_4_, 1.18; EDTA, 0.026; and D-glucose, 5.5 mM. Segments were cleaned of connective tissue and mounted on two 40 µm wires in a wire myograph (Danish Myo Technology A/S, Denmark). Segments were bathed in PSS, heated to 37°C and continuously gassed with 95% O_2_ and 5% CO_2_. The passive tension-internal circumference relationship (IC_100_) was determined by incremental increases in tension to achieve an internal circumference equivalent to a transmural pressure of 100 mmHg (using the Laplace relationship), and the arteries were set to a diameter equivalent to 0.9×IC_100_. Functional integrity of the smooth muscle was assessed with four two minute washes with 125 mM KPSS solution (PSS solution with an equimolar substitution of KCl for NaCl) [21]. Vessels failing to produce an active tension equivalent to 13.3 kPa were discarded from the study. Following normalisation, cumulative concentration response curves were obtained for NA (10 nM–100 µM). Segments were then preconstricted with 0.1 µM of the thromboxane mimetic U46619 and cumulative concentration-response curves (CRCs) to the endothelium-dependent vasodilator ACh; (0.1 nM–10 µM) and the NO donor, SNP (0.1 nM–30 µM) were performed. The dilator response was calculated as the percentage relaxation of the NE-induced tone and is expressed as the % relaxation. To determine the relative contribution of NO to ACh-mediated vasodilation, responses to ACh were repeated in the presence of the NO synthase inhibitor N^ω^-nitro-L-arginine methyl ester (L-NAME; 100 µM) in a sub-set of femoral arteries from all four offspring groups.

### Femoral Artery NO Levels

Basal and ACh (1 µM) stimulated NO release in femoral arteries was assessed using 4,5-diaminofluorescein diacetate (DAF-FM) and imaged using a Leica SP5 confocal microscope (excitation 488 nm, emission 515–530 nm) [22]. Values were corrected for background and expressed as arbitrary units (AU) and normalised to C/C offspring.

### Femoral Artery eNOS mRNA Expression

Expression of eNOS mRNA in femoral arteries was analysed by real-time PCR and normalised to β-actin as described previously [23]. The eNOS probes and primers were purchased from Eurogentec UK and were as follows: forward primer, 5′-GGAAATGTCAGGCCCGTACA-3′; reverse primer, 5′-GTCTGAGCAGGAGACA CTGTTGA-3′; probe, 5′-FAM-TGAGCAGCACAAGAGCTACAAAATCCGA-TAM RA-3′. Each sample was assayed in duplicate in a single 96-well plate and a mean copy number calculated.

### ROS Generation in Femoral Arteries and Skeletal Muscle

Superoxide generation in fresh femoral artery segments and frozen sections of vastus muscle was assessed using the redox-sensitive dye dihydroethidium (DHE) (5 µM (femoral vessels; 10 µM vastus muscle for 45 mins in PBS) as previously described [24]. Tissues were then fixed in 2% paraformaldehyde and visualised in the confocal microscope using a x20 oil immersion objective. The images were quantified using Image J software (NIH, USA). Values were corrected for background and intensities expressed as arbitrary units (AU). DHE specificity for superoxide was determined in the presence of superoxide dismutase (SOD) (500 µL/ml) (data not shown).

### Vascular Nox2 and HO-1 Protein Expression

Protein expression of Nox2 and HO-1 in aortic homogenates from all four dietary groups of animals at 30 weeks was measured by immunoblotting [25]. Protein content in aortic lysates was determined using the bicinchoninic acid assay (BCA, Pierce), and lysates were subjected to sodium dodecyl sulphate (SDS) gel electrophoresis and immunoblotted with antibodies against HO-1 and Nox2 with α-tubulin used as a loading control. Enhanced chemiluminescence was used to visualize bands on autoradiographic film (Amersham, UK) and which were quantified using Image J software (NIH, USA).

### Assessment of ROS Generation in Liver

ROS generation was assessed using enhanced chemiluminescence in liver (2 mm^3^ segments) incubated with lucigenin (5 µM) and NADPH (100 µM) [26]. Luminescence (averaged over 10 min) was expressed as mean light units (MLU)/mg dry weight of tissue (n = 3–4 segments per animal).

### Microarray Analysis in Liver

Total RNA was extracted from liver tissue using TRIzol® reagent (Invitrogen, UK). RNA was further purified using RNA clean up Kit™ (Zymo Research). Total RNA from each male offspring group was pooled (1 µg of total RNA from each sample, n = 6 each group) and sent for whole genome gene expression analysis (Nimblegen, Iceland). ArrayStar (DNASTAR) software was used to compare arbitrary expression values from each group exposed to a HF diet (C/HF, HF/C and HF/HF) against those from the control group (C/C), and a fold difference value was generated for each gene. Changes were considered marked if the fold difference was >1.7 and genes within a pathway showed a similar pattern of expression.

### Statistical Analysis

All statistical analyses were performed using SPSS for Windows version 18.0 (SPSS, Chicago, IL, USA). Data from offspring from the four dietary groups (C/C, HF/C, C/HF and HF/HF) studied at 15 or 30 weeks of age were analysed using analysis of variance (ANOVA) with maternal with maternal diet, offspring diet and age as between subject factors. Specific comparisons between HF-fed offspring groups and C/C control offspring at each age point were made using *post hoc* Dunnett’s or Bonferroni multiple comparison tests where appropriate. Relationships between variables were assessed by linear regression. All data are expressed as mean ± S.E.M. Statistical significance was accepted if *p*<0.05. Investigators were blinded to the dietary group at all points of the study.

## Results

### Maternal Body Weight and Body Fat

Dams fed a HF diet for 4–6 weeks prior to mating, during gestation and lactation were 25% heavier (C, 26.0±0.93 g; HF, 31.9±0.83 g) and had 130% more body fat (C, 8.3±0.3%; HF, 19±2%) at weaning compared with chow fed controls (n = 10 per group, p<0.001).

### Impact of Maternal HF-feeding on Offspring Body Weight, Body Fat and Systolic Blood Pressure

Offspring body weight measured in the four offspring dietary groups at 15 or 30 weeks of age is shown in Figure 1A. There were significant effects of maternal dietary fat and age on offspring body weight (Table 2). At 15 weeks of age body weight was significantly increased in offspring groups exposed to post-weaning HF (C/HF and HF/HF, *p*<0.001) but not pre-weaning (HF/C) compared to C/C control offspring. At 30 weeks of age offspring body weight was increased in all offspring groups exposed to HF, compared to C/C control offspring (HF/C, C/HF and HF/HF vs. C/C, all *p*<0.001). Additionally, body fat mass measured at 30 weeks of age was significantly increased in all HF fed offspring (HF/C, C/HF and HF/HF) (maternal diet, F = 48.5, *p*<0.0001; offspring diet, F = 54.1, *p*<0.0001; maternal diet*offspring diet, F = 29.2, *p*<0.0001) (please see *Figure S2*).

Offspring systolic blood pressure was significantly influenced by maternal diet, offspring diet and age (Table 2). At 15 weeks of age offspring systolic blood pressure was increased in C/HF and HF/HF offspring groups, compared to C/C control offspring (*p*<0.001) (Figure 1B). At 30 weeks of age systolic blood pressure was increased in HF/C, C/HF and HF/HF offspring compared to age matched C/C control offspring (all *p*<0.001). Systolic blood pressure in HF/C offspring did not differ significantly from that of C/HF offspring (Figure 1B). There was also a significant interaction between maternal and offspring post-weaning diet at 30 weeks of age (F = 10.7, *p* = 0.003).

### Impact of Maternal HF-feeding on Offspring Femoral Vascular Reactivity

Vasoconstriction to NA in femoral artery measured in male offspring across the four offspring dietary groups was increased by age (*p*<0.0001) but was not influenced by maternal or offspring diet (Figure 2A and B).

At 15 weeks of age ACh elicited a significant vasorelaxation in the femoral artery of all dietary groups compared (Figure 3A). The ACh-mediated vasorelaxation measured in HF/C and HF/HF offspring was lower than that in C/C offspring at 15 weeks (*p*<0.05). At 30 weeks of age maximal vasorelaxation to ACh was significantly reduced in HF/C, C/HF, HF/HF offspring groups compared to C/C (*p*<0.05, *p*<0.001, *p*<0.001, respectively) (Figure 3B). The vasorelaxation measured in HF/C offspring at 30 weeks of age was not significantly different from that in C/HF offspring (*p*>0.05, Mann Whitney test). Age independently influenced endothelium-dependent vasorelaxation to ACh (Table 2) and comparison of the maximal relaxation to ACh at the two age points studied showed a significant reduction in maximal relaxation in all dietary groups (C/C, HF/C, C/HF and HF/HF 15 vs. 30 weeks; Mann Whitney test, all *p*<0.05).

The NO-component of the ACh-induced vasorelaxation was investigated using L-NAME (100 µM) in arteries from the four offspring dietary groups at 15 weeks only as the lack of a robust ACh-induced vasorelaxation in arteries from HF-fed offspring at 30 weeks precluded investigation of the NO-component using L-NAME at the later age point. At 15 weeks of age, pre-incubation with L-NAME attenuated the response to ACh in all four dietary groups (all *p*<0.01, L-NAME vs. naïve preparations) (Figure 4) (please see *Figure S3* for full dose response curves). The relative contribution of NO (as blocked by L-NAME) to the ACh-induced vasorelaxation did not differ significantly across the four dietary groups (C/C, 79±10%; HF/C, 70±15%; C/HF, 85±10%; HF/HF, 98±2%).

The NO-donor SNP produced a concentration-dependent vasorelaxation that was similar in all offspring groups at 15 weeks (% maximal response; C/C, 60.7±2.9, n = 6; HF/C, 48.5±5.0, n = 4; C/HF, 62.4±11.1, n = 5; HF/HF, 39.4±4.0, n = 7) and at 30 weeks of age (% maximal response; C/C, 42.9±2.7, n = 8; HF/C, 33.9±5.1, n = 5; C/HF, 41.8±5.8, n = 8; HF/HF, 33.4±2.7, n = 7; *Figure S4*).

### Impact of Maternal HF-feeding on Offspring NO Production and eNOS mRNA Levels in the Femoral Artery

Basal NO production in femoral arteries from control (C/C) male offspring was 1.7±0.2 AU and 9.6±1.9 AU at 15 weeks and 30 weeks of age, respectively (n = 3 per group). Basal NO production was significantly reduced in offspring fed HF post weaning (C/HF and HF/HF) compared with their age matched C/C controls (p<0.05) at 15 weeks (Figure 5A). At 30 weeks all HF-fed offspring groups (HF/C, C/HF and HF/HF) showed a reduced basal NO-production compared with their age matched C/C controls (*p*<0.001) (Figure 5A). ACh-induced NO production in C/C femoral arteries at 15 weeks and 30 weeks of age was 3.3±0.3 AU and 18.3±0.9 AU, respectively (n = 3 per group), (*p*<0.001; C/C basal vs. C/C ACh-induced 15 and 30 weeks) and was reduced in all HF-fed offspring groups at both 15 and 30 weeks of age (*p*<0.05) (Figure 5B) (please see *Figure S5* for illustrative confocal images of DAF staining at baseline and in the presence of ACh at 30 weeks).There was a strong correlation between basal and ACh-induced NO production across the four offspring groups at 15 and 30 weeks of age (r = 0.91, *p*<0.0001). Additionally, the reduction in ACh-induced NO production in HF/C offspring did not differ significantly from that in offspring fed a HF diet post weaning (C/HF). Maternal diet, offspring diet and age all influenced basal and ACh-induced NO-production in the femoral artery in the four dietary groups (Table 2). There was also a significant interaction between maternal and post-weaning offspring diet to influence ACh-induced NO production (F = 6.3, *p* = 0.022). Levels of eNOS mRNA in the femoral artery were found to be similar between the four dietary groups at 30 weeks of age (C/C, 1.16±0.15; HF/C, 1.17±0.12; C/HF, 1.07±0.13; HF/HF 1.13±0.14; n = 6 per group).

### Impact of Maternal HF-feeding on Offspring Redox Regulation

DHE staining in the femoral artery was increased similarly in all HF-fed offspring (*p*<0.05 vs. C/C) at 15 weeks and was significantly influenced by offspring diet (Figure 6A and Table 2). DHE staining measured in the vastus lateralis muscle (supplied by the femoral artery) at 30 weeks was greater in all high fat fed groups compared to controls (Figure 6B) and was influenced by offspring diet (F = 11.5, *p* = 0.004; interaction maternal diet×offspring diet F = 25.2, *p*<0.0001).

NADPH-dependent superoxide generation assessed by lucigenin chemiluminescence in the liver at 15 weeks was significantly increased in HF/C offspring compared to age matched chow fed offspring (*p*<0.05, HF/C vs. C/C) (Figure 7). While NADPH-dependent superoxide generation more than doubled in C/C offspring at 30 weeks (p = 0.03, vs. C/C 15 weeks), there was no further increase in hepatic superoxide generation in 30 week old offspring over that measured at 15 weeks in offspring exposed to the HF diet (HF/C, C/HF, HF/HF). Further, at 30 weeks hepatic superoxide generation did not differ significantly between the pre- and post- weaning HF groups. In order to identify candidate genes that might account for the complexity and extent of the reduced bioavailability of NO, hepatic RNA was isolated from 15 and 30 week old animals from all four dietary groups and gene expression changes analysed using microarray. Microarray analysis identified patterns of altered regulation (>1.7 fold change vs. C/C) in a number of genes involved in the ROS/NOS and inflammatory pathways in the liver of male mice including NADPH oxidase 2 (Nox2) and the stress response enzyme heme oxygenase-1 (HO-1) (see *Table S1* for details). Vascular Nox-2 protein expression did not differ between the offspring groups at 30 weeks; however HO-1 protein expression in the aorta of the sub group of offspring studied at 30 weeks showed only a trend (*p* = 0.068) towards a reduction in HF fed offspring vs. C/C (Figure 8).

## Discussion

This study demonstrates that maternal intake of fat during pregnancy and lactation, at a level that approximates to the upper end of female human fat intake, leads to persistent increases in body weight and fat accumulation and raised systolic blood pressure in male offspring. Our data provide clear evidence of an attenuated endothelial NO-mediated vasorelaxation and a decreased NO bioavailability both under basal conditions and during exposure to ACh. They further suggest that the reduced vasorelaxation is in part attributable to a perturbed redox status in the offspring. The adverse vascular and metabolic phenotype of offspring exposed to a high fat diet during gestation and suckling was comparable to that of offspring exposed to a high fat diet during adult life alone; and was exacerbated by age.

The fat intake of the mouse dams for 4–6 weeks pre-pregnancy and during pregnancy and suckling was at a level that approximates to the upper end of female human fat intake [27] and reflects a human ‘obesogenic’ diet. The gain in body weight and body fat by the mouse dams fed a HF diet (45% kcal fat) is consistent with that seen in other rodent models of maternal over-nutrition (reviewed in Ainge *et al*. [28] and Li *et al.* [29]) and recently reported in mice fed a diet high in fat or a palatable obesogenic diet. The adult male offspring of dams fed a HF diet displayed increased body weight and adiposity, impaired glucose tolerance and increased plasma insulin levels, and raised systolic blood pressure similar to that we have reported previously in male and female mice offspring of dams fed a HF diet supplemented with 18% (w/w) lard [14]. However, our present novel findings in offspring studied at 15 and 30 weeks of age reveal that while post weaning HF consumption appears to have a greater initial impact on the younger offspring body weight and systolic blood, as they age the effects of maternal HF consumption and a post-weaning HF diet on their phenotype appear to converge.

### Vascular Relaxation is Affected by Maternal HF Diet

Reports on the effects of total dietary fat and dietary fatty acid composition on vascular function and blood pressure are inconclusive [30]; as are those on the mechanisms underlying a reduced vasodilator capacity (see [31], [32] for reviews). In order to investigate the effects of a maternal and/or offspring high fat diet on vascular function and NO bioavailability, we assessed perturbations in endothelium-mediated vasorelaxation and factors that might influence it in the femoral artery. Femoral artery endothelium-dependent vasorelaxation to ACh is largely mediated by NO as confirmed by our finding that more than two thirds of the ACh-induced vasorelaxation was blocked by L-NAME in arteries from C/C offspring. At 15 weeks, arteries from HF/C and HF/HF offspring showed an approximately 50% attenuation in ACh-mediated relaxation compared with C/C offspring, indicative of an impact of exposure to excess fats during early development on the NO-dependent endothelial signaling pathway. An impact of a post-weaning offspring high fat (C/HF) on ACh-mediated relaxation was not evident until 30 weeks of age at which time all dietary HF-fed offspring groups showed a reduced ACh-mediated relaxation. Interestingly, while at 30 weeks of age, an attenuated ACh-mediated relaxation was associated will a reduction in NO bioavailability and an increase in systolic blood pressure, at 15 weeks there was no increase in blood pressure in HF/C and HF/HF in spite of a reduction in NO bioavailability. We did not find any increase in NA-induced activity in these vessels indicative of an enhanced constrictor tone which might offset NO-mediated dilatation and increase blood pressure. Thus it is possible that either alteration in femoral artery tone has little effect on peripheral resistance and hence blood pressure or that extended intake of a fatty diet and/or increasing body fat results in a disruption of other dilator pathways.

In cardiovascular pathologies characterized by a reduced NO-mediated vasorelaxation, a compensatory up-regulation of endothelium derived hyperpolarizing factors (EDHFs) may serve to sustain dilatation and maintain tissue perfusion [33]. Evidence for an up-regulation of EDHF-mediated relaxation is seen in small resistance arteries and arterioles from animal models of diet-induced obesity [34]–[36], hypercholesterolemia [37], hypertension [38], diabetes [39] and in ApoE and LDL receptor-deficient mice fed a high fat diet in adult life [40]. It has also been reported in studies on mesenteric arteries from offspring in other models of developmental priming [41]–[43]. We saw no compensatory up-regulation of a non-NO pathway in the present study using the femoral artery.

*Perturbation of offspring NO bioavailability and redox status by maternal high fat diet.*

To address whether the effects of maternal and offspring diet on vasorelaxation were due to a reduced NO bioavailability, we assessed basal and ACh-stimulated NO release using DAF in fresh, excised femoral arteries. The impact of a high fat diet was most marked at 30 weeks when basal and ACh-stimulated NO release was reduced by over 70% in all high fat fed groups (HF/C, C/HF, HF/HF) compared with chow fed control offspring. Basal and ACh-stimulated NO release was similarly reduced, but to a lesser extent (∼30%), in high fat fed offspring at 15 weeks of age. We found no difference in the level of eNOS mRNA in the femoral artery across the offspring groups. Thus it is unlikely that reduced expression at the mRNA level of the constitutively produced NOS influences NO generation in the femoral artery of the primed offspring. It is however possible that the high fat diet modulates tetrahydrobiopterin (BH_4_) levels as has been reported in a model of maternal nutrient restriction [44] and/or the activity of other NOS enzymes influenced by the maternal diet.

A primary mechanism for a decrease in NO bioavailability is its rapid degradation by interaction with superoxide ion (O_2_^−^) to form peroxynitrite (ONOO^−^) [10]. Increased oxidative stress and impaired vascular function in obesity is well documented [8] and reduced NO bioavailability and increased ROS generation are reported to be key mechanisms mediating the age-related decline of endothelium-dependent relaxation [45], [46]. Pregnancy is also associated with increased oxidative stress and lower levels of antioxidant enzymes measured in plasma are associated with both small and large for gestational age babies [47]. Additionally, gestational obesity (as modelled in our mouse dams) can further lead to unbalanced maternal oxidant/antioxidant status and enhance oxidative stress in the fetus [48] and offspring [19], [49]. Cafeteria-fed rat dams show raised plasma protein carbonyl and lower total antioxidant status, with similar changes reported in the offspring at birth and during adulthood [49]. In our model, increased DHE staining in all HF-fed offspring groups is consistent with an increase in O_2_^−.^ generation and suggests that decrements in vascular function and NO-bioavailability are in part due to an increased ROS production.

NADPH oxidases are an important source of O_2_^−.^ under physiological conditions and participate in the development of vascular disease [50]. Augmented Nox2 expression has been linked to aortic [51] and cerebral vascular [52] dysfunction in male apolipoprotein E-null (ApoE^−/−^) mice maintained on a HF (21%) diet from 5 weeks of age, and inhibition of Nox2 reduces the hypertension [53]. We saw an increase in hepatic NADPH-dependent O_2_^−^ generation in HF/C offspring at 15 weeks that was of similar magnitude to the O_2_^−^ generation measured in all offspring groups at 30 weeks. The early increase was consistent with that seen by Franco *et al.* [54] in male rat offspring from under-nourished dams and possibly indicative of an accelerated or ‘early aging’ in the programmed offspirng. We were unable to demonstrate an impact of either maternal or offspring diet on Nox2 protein expression in the aorta of offspring measured at 30 weeks of age. One explanation for this is that in our model of developmental priming through a maternal HF diet, prolonged activation of NADPH oxidase in early life may lead to depletion of intracellular NADPH and decreased NADPH oxidase activity other than Nox2 expression [55].

Findings relating to antioxidant status in human obesity and in animal models of developmental priming remain contradictory [7], [20]. HO-1 is expressed at low basal levels in the vasculature and as well as being a potent endogenous antioxidant is directly involved in the regulation of vascular tone via the generation of carbon monoxide (CO) [56]. Our observation of a trend towards reduced HO-1 in vascular tissue and the liver is consistent with reports of a reduced antioxidant scavenging capacity in the plasma [49] and liver [19] of programmed adult offspring. A reduction in HO-1 expression may thus reflect not only an imbalance in oxidant/antioxidant status, but may also contribute to the reduced vasodilator capacity observed in our animals.

### Interactions between Maternal Diet, Offspring Diet and Age

Previous studies in six month-old female rats exposed to a maternal diet rich in animal fat prenatally and during early postnatal life have shown that, while these offspring go on to develop conditions resembling the human metabolic syndrome, endothelial dysfunction (excluding blood pressure) was ameliorated if the offspring were raised on a similar fat-rich diet to their dams [57]. This was not so in the current study, where there was interaction between maternal and post-weaning diet to amplify vascular dysfunction in the adult offspring. It is possible that the difference in species, sex, and proportion and nature of the fat and carbohydrate content of the maternal and post-weaning diet may contribute to the differences in outcome. We have previously shown in our maternal HF feeding model that intra-uterine and pre-weaning high dietary fat exposure enhances vulnerability to a ‘second hit’ from high post-weaning dietary fat consumption in the offspring, to promote progression of liver disease [18]. Thus it is plausible that a similar priming of endothelial signalling pathways occurs and that this is induced to greater effect in offspring exposed to both adverse developmental and adult environments. Additionally, while our data strongly support an interaction between maternal and offspring HF diet to worsen vascular function, we can only speculate as to whether the additional impact of the post-weaning HF diet on endothelium-dependent relaxation and redox regulation is mediated by an effect of extended intake of a fatty diet, increasing body fat and metabolic stress (manifest as fatty liver disease and impaired glucose metabolism) in the adult offspring [18]. Further, with our current study design we cannot determine which component of the maternal diet and/or maternal phenotype (e.g. obesity) causes these effects in the offspring [28].

In our mouse model, maternal diet and offspring diet also interacted with age to attenuate vascular function in the adult offspring. Thus, ageing-induced reductions in NO production and increase in ROS [58], [59] associated with altered activity of NADPH oxidase may contribute to the decline in NO-mediated vasorelaxation and subsequent increases in systolic blood pressure that we observe in all HF-fed dietary groups at 30 weeks of age. Our findings are consistent with other reports showing that age impairs endothelial function in conduit and resistance arteries in human and animal models (for recent review see [58]) and supported by recent findings by Collins et al. that ageing enhances the metabolic and vascular effects of a high fat diet in low-density lipoprotein receptor knockout (LDLr−/−) mice [60].

### Perspectives

Our data demonstrate that exposure to excess fat during pregnancy and suckling reduces endothelium NO-dependent vasorelaxation through an unbalanced production or scavenging of ROS in the offspring (Figure 9). Such an imbalance in NO- and ROS-mediated signalling has been shown to contribute to the development and progression of cardiovascular disease, including coronary artery disease and hypertension, and insulin resistance in obesity and diabetes. There have been few investigations of associations between over-nutrition/obesity in pregnant women and CV risk in their children [61], [62] and the extent to which developmental ‘priming’ may predispose to cardio-metabolic disease risk in children of over-nourished/obese mothers has yet to be fully elucidated. Our findings emphasize the importance of a balanced diet during pregnancy and lactation and provide a framework for understanding the mechanistic processes by which priming of risk for cardiovascular and metabolic disease may occur during early life.

## Supporting Information

Figure S1 **Schematic representation of high fat dietary model.**
(DOCX)

Figure S2 **Offspring body fat from the four dietary groups measured at 30 weeks of age.**
(DOCX)

Figure S3 **Dose response curves of offspring femoral arteries to ACh ± LNAME.**
(DOCX)

Figure S4 **Cumulative dose response curves of offspring femoral arteries to the NO-donor SNP.**
(DOCX)

Figure S5 **Images of NO bioavailability using DAF staining in femoral arteries from four offspring groups at 30 weeks of age.**
(TIF)

Table S1 **Microarray-generated relative expression of genes involved in NOS signaling and REDOX balance measured in liver from male mouse offspring.**
(DOCX)
