# Supplementary material for: Interaction between Maternal and Offspring Diet to Impair Vascular Function and Oxidative Balance in High Fat Fed Male Mice
Source: PLoS One. 2012 Dec 5;7(12):e50671. doi: 10.1371/journal.pone.0050671 (PMC3515587; doi:10.1371/journal.pone.0050671)
Supplement: Figure S1 — Schematic representation of high fat dietary model. (DOCX) [file pone.0050671.s001.docx]

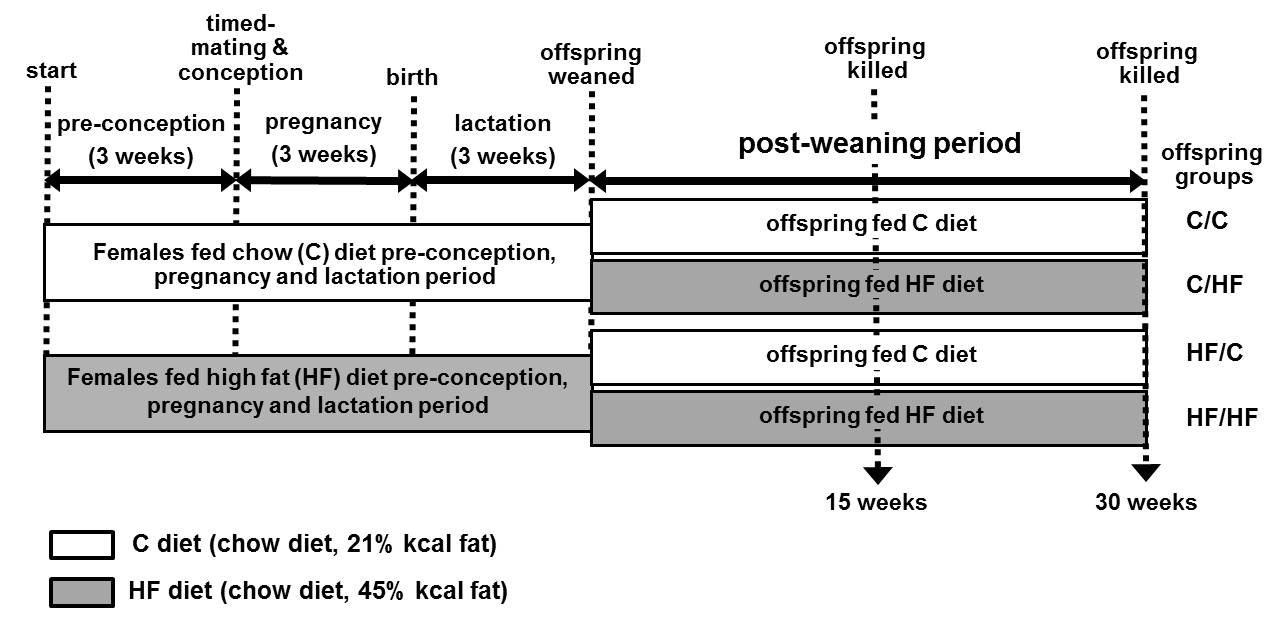


**Figure S1.** Schematic representation of high fat dietary model. Female C57BL6J mice (n=20) were fed either standard chow (C, 21% kcal fat) or saturated fat (HF, 45% kcal fat) for 4 weeks prior to conception, during gestation and lactation. At 3 weeks of age male offspring were weaned and assigned to either HF or C diet, generating 4 experimental groups (n = 8 per group): C/C, C/HF, HF/C and HF/HF. At 15 and 30 weeks of age, male offspring were killed by an overdose of anaesthetic (isofluorane) and cervical dislocation.
