# Supplementary material for: Interaction between Maternal and Offspring Diet to Impair Vascular Function and Oxidative Balance in High Fat Fed Male Mice
Source: PLoS One. 2012 Dec 5;7(12):e50671. doi: 10.1371/journal.pone.0050671 (PMC3515587; doi:10.1371/journal.pone.0050671)
Supplement: Figure S2 — Offspring body fat from the four dietary groups measured at 30 weeks of age. (DOCX) [file pone.0050671.s002.docx]

**Figure S2.** Offspring body fat (white adipose tissue, WAT) measured at 30 weeks of age. The fat pads that were dissected and weighed include the intrascapular, retroperitoneal, inguinal, perirenal and gonadal fat depot. Body fat was expressed as the percent of body weight accounted for as fat. Data are mean ± SEM (n=7 per group).**p*<0.01
