# Supplementary material for: Interaction between Maternal and Offspring Diet to Impair Vascular Function and Oxidative Balance in High Fat Fed Male Mice
Source: PLoS One. 2012 Dec 5;7(12):e50671. doi: 10.1371/journal.pone.0050671 (PMC3515587; doi:10.1371/journal.pone.0050671)
Supplement: Figure S3 — Dose response curves of offspring femoral arteries to ACh ± LNAME. (DOCX) [file pone.0050671.s003.docx]

**Figure S3.** Femoral dilation to ACh alone or in the presence (closed circles) and absence of N^ω^-nitro-L-arginine methyl ester (L-NAME) (100 μM) in the four male offspring groups at 15 weeks of age. Data are mean ±SEM of n=4-6 per dietary group.
