# Supplementary material for: Interaction between Maternal and Offspring Diet to Impair Vascular Function and Oxidative Balance in High Fat Fed Male Mice
Source: PLoS One. 2012 Dec 5;7(12):e50671. doi: 10.1371/journal.pone.0050671 (PMC3515587; doi:10.1371/journal.pone.0050671)
Supplement: Figure S4 — Cumulative dose response curves of offspring femoral arteries to the NO-donor SNP. (DOCX) [file pone.0050671.s004.docx]

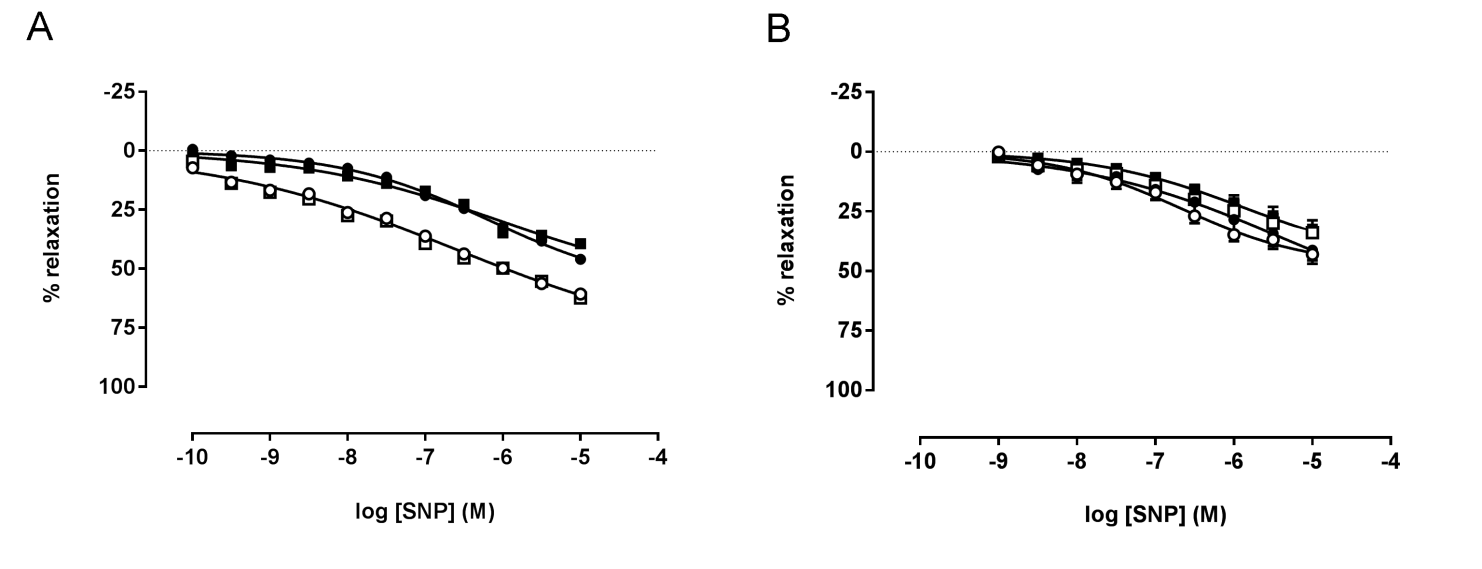


**Figure S4** Cumulative dose response curves to the NO-donor sodium nitroprusside (SNP) measured in the femoral artery of male mice offspring from four dietary groups at (A) 15 weeks (⭘ C/C n= 6, ⚫ HF/C n=6, 🞎 C/HF n=5, ◼ HF/HF n= 7) and (B) 30 weeks of age. (⭘ C/C n= 8, ⚫ HF/C n=5, 🞎 C/HF n=8, ◼ HF/HF n=7). Data are mean ± SEM.
