# Supplementary figures and images for: Interaction between Maternal and Offspring Diet to Impair Vascular Function and Oxidative Balance in High Fat Fed Male Mice
Source: PLoS One. 2012 Dec 5;7(12):e50671. doi: 10.1371/journal.pone.0050671 (PMC3515587; doi:10.1371/journal.pone.0050671)

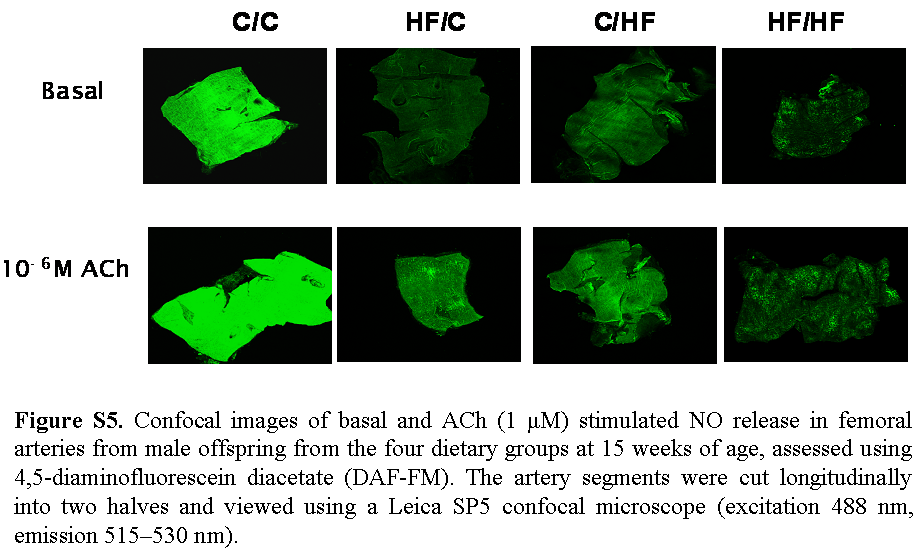

Supplement: Figure S5 — Images of NO bioavailability using DAF staining in femoral arteries from four offspring groups at 30 weeks of age. (TIF) [file pone.0050671.s005.tif]
