# Supplementary material for: Interaction between Maternal and Offspring Diet to Impair Vascular Function and Oxidative Balance in High Fat Fed Male Mice
Source: PLoS One. 2012 Dec 5;7(12):e50671. doi: 10.1371/journal.pone.0050671 (PMC3515587; doi:10.1371/journal.pone.0050671)
Supplement: Table S1 — Microarray-generated relative expression of genes involved in NOS signaling and REDOX balance measured in liver from male mouse offspring. (DOCX) [file pone.0050671.s006.docx]

**Table S1.** Microarray-generated relative expression of genes involved in NOS signaling and REDOX balance measured in liver from male mouse offspring of dams fed a diet high in saturated fat (HF) or standard chow (C) pre-conception and throughout pregnancy and lactation and then continued post weaning on the same diet as their dams or placed onto the alternative diet to give four dietary groups (C/C, HF/C, C/HF and HF/HF). Offspring were studied at 15 or 30 weeks of age. Total RNA was extracted from liver tissue using TRIzol® reagent (Invitrogen, UK). RNA was further purified using RNA clean up Kit™ (Zymo Research). Total RNA from each male offspring group was pooled (1 μg of total RNA from each sample, n=6 each group) and sent for whole genome gene expression analysis (Nimblegen, Iceland). ArrayStar (DNASTAR) software was used to compare arbitrary expression values from each group exposed to a HF diet (C/HF, HF/C and HF/HF) against those from the control group (C/C), and a fold difference value was generated for each gene.

|  |  | 15 weeks |  |  | 30 weeks |  |
| --- | --- | --- | --- | --- | --- | --- |
| NOS Signalling | HF/C | C/HF | HF/HF | HF/C | C/HF | HF/HF |
| iNOS | +1.5 | +2.3 | +1.3 | -1.0 | +1.0 | +1.4 |
| eNOS | +1.2 | +1.3 | +1.1 | -1.1 | +1.3 | +1.5 |
| Cav-1 | +1.0 | +1.1 | +1.1 | -1.1 | -1.8 | -1.6 |
| GS | +1.8 | +2.3 | +2.8 | +1.3 | +1.5 | +1.5 |
| Antioxidant Enzymes |  |  |  |  |  |  |
| HO-1 | +1.7 | +2.1 | +1.4 | +1.1 | +1.8 | +1.2 |
| NQO1 | +1.2 | +1.9 | +2.8 | +1.3 | +1.7 | +1.5 |
| GSTk1 | +1.7 | +2.5 | +1.4 | +3.7 | +1.0 | +1.9 |
| MnSOD | +1.6 | +2.3 | +2.1 | +1.4 | +1.1 | +1.1 |
| Cu/Zn SOD | -1.0 | -1.2 | -1.1 | -1.2 | -1.1 | -1.1 |
| EC-SOD | -1.7 | -1.1 | -1.0 | -1.1 | +1.9 | +1.5 |
| Nrf2 | +1.2 | +1.2 | +1.4 | +1.3 | +1.2 | +1.2 |
| GPx-1 | -1.0 | -1.0 | -1.1 | +1.1 | +1.1 | +1.1 |
| GPx-4 | +1.2 | +1.6 | +1.1 | +1.1 | -1.2 | +1.5 |
| Junc (AP-1) | +1.2 | +2.3 | +1.8 | +1.4 | +1.5 | +2.4 |
| NADPH Oxidases |  |  |  |  |  |  |
| NOX2 | +3.5 | +2.9 | +5.1 | +1.1 | +1.5 | +1.7 |
| NOX3 | -1.3 | -2.0 | -1.8 | +1.9 | +1.3 | -2.7 |
| NOX4 | +1.5 | -1.1 | +1.3 | +1.0 | -1.1 | -1.1 |
| Other Regulators of NADPH Oxidases |  |  |  |  |  |  |
| PPAR-γ | +1.2 | +1.2 | +2.0 | +1.3 | +1.4 | +1.9 |
| TGFβ-1 | +3.0 | +2.5 | +3.7 | -1.0 | +1.1 | +1.1 |
| TGFβ-i | +1.6 | +2.0 | +3.0 | +1.3 | +2.2 | +2.0 |
